# Incidence, Microbiological Profile and Risk Factors of Healthcare-Associated Infections in Intensive Care Units: A 10 Year Observation in a Provincial Hospital in Southern Poland

**DOI:** 10.3390/ijerph15010112

**Published:** 2018-01-11

**Authors:** Małgorzata Kołpa, Marta Wałaszek, Agnieszka Gniadek, Zdzisław Wolak, Wiesław Dobroś

**Affiliations:** 1 The Institute of Health Sciences, State Higher Vocational School in Tarnów, ul. Mickiewicza 8, 33-100 Tarnów, Poland; malgorzatakolpa@interia.pl (M.K.); mz.walaszek@gmail.com (M.W.); zdzich_w@interia.pl (Z.W.); wdobros@wp.pl (W.D.); 2 Department of Nursing Management and Epidemiology Nursing, Faculty of Health Sciences, Jagiellonian University Medical College, ul. Kopernika 25, 31-501 Kraków, Poland

**Keywords:** healthcare-associated infections, hospital infection intensive care unit, bloodstream infection urinary tract infection, ventilator-associated pneumonia, antibiotic resistance, *Acinetobacter baumannii*

## Abstract

Healthcare-associated infections (HAIs) occurring in patients treated in an intensive care unit (ICU) are serious complications in the treatment process. Aetiological factors of these infections can have an impact on treatment effects, treatment duration and mortality. The aim of the study was to determine the prevalence and microbiological profile of HAIs in patients hospitalized in an ICU over a span of 10 years. The active surveillance method was used to detect HAIs in adult patients who spent over 48 h in a general ICU ward located in southern Poland between 2007 and 2016. The study was conducted in compliance with the methodology recommended by the Healthcare-associated Infections Surveillance Network (HAI-Net) of the European Centre for Disease Prevention and Control (ECDC). During the 10 years of the study, 1849 patients hospitalized in an ICU for a total of 17,599 days acquired 510 with overall HAIs rates of 27.6% and 29.0% infections per 1000 ICU days. Intubation-associated pneumonia (IAP) posed the greatest risk (15.2 per 1000 ventilator days), followed by CLA-BSI (8.0 per 1000 catheter days) and CA-UTI (3.0 per 1000 catheter days). The most common isolated microorganism was *Acinetobacter baumannii* (25%) followed by Coagulaase-negativ staphylococci (15%), *Escherichia coli* (9%), *Pseudomonas aeruginosa* (8%), *Klebsiella pneumoniae* (7%), *Candida albicans* (6%). *Acinetobacter baumannii* in 87% and were classified as extensive-drug resistant (XDR). In summary, in ICU patients pneumonia and bloodstream infections were the most frequently found. *Acinetobacter baumannii* strains were most often isolated from clinical materials taken from HAI patients and showed resistance to many groups of antibiotics. A trend of increasing resistance of *Acinetobacter baumannii* to carbapenems was observed.

## 1. Introduction

According to multiple sources, between 20% and 50% of healthcare-associated infections (HAIs) occur in the intensive care unit (ICU). In 2007, 39% of patients in European countries were affected by these infections [1]. In Poland HAIs were detected in 24–45% of ICU patients [2,3]. Meta-analysis of HAIs showed that a HAIs affected 30% of patients [4]. In Italy frequency of HAIs was lower (16%) [5]. These infections presented in the most dangerous and sometimes life-threatening forms of infection, such as pneumonia, bloodstream infection, and urinary tract infection; therefore, surveillance of these types of infections is important in the ICU [6,7]. The prevention of these infections is correlated with high treatment costs and longer hospitalization times [8]. Among healthcare-associated infections, PN is the most common and accounts for between 30% and 50% of all infections [6,8,9,10,11]. Epidemiological studies indicate that bloodstream infections are low frequently found and their prevalence ranges from 4% to 20% [6,8,10,11]. Among HAIs, the prevalence of urinary tract infections ranges from 3% to 7% [8,10,11]. Studies by Gordats et al. [10] showed that, among individuals with reduced immunity, which included ICU patients, other forms of HAIs also appeared.

The development of HAIs can be facilitated by the ICU environment, medical procedures applied to treat the patient and, above all, the patient’s general health. Intensive care units treat severely ill patients whose underlying disease and coexisting diseases may contribute to the development of healthcare-associated infections [10,12]. The need to perform many diagnostic and treatment activities on patients results in the use and long-term maintenance of invasive devices, such as central vascular lines (CVC), intubation tubes, and urethral catheters (which may result in the removal of the natural protective barrier against infections [13]. Patients’ stays in the ICU are often preceded by hospitalization in other hospital wards, during which there are often problems with the treatment of such patients. During the patient’s stay in hospital (regardless of the ward in which they are hospitalized), the bacterial physiological microbiota are replaced with the hospital microbiota typical of a given ward. In addition, long-term antibiotic treatment leads to the selection of sensitive microorganisms in the patient’s organisms, which may facilitate a conquest of the organism by pathogenic microorganisms (also by those coming from the environment) with a proven mechanism of resistance to antibiotics [13,14]. Therefore, it is extremely important to undertake actions that will at least protect patients from the negative health effects of microorganisms coming from a hospital environment; this is especially true for patients in intensive care units, where the invasiveness of treatments and reduced immunity of the patient will predispose them to an increased number of colonizations and infections. The results of the analysis demonstrated below are among few such studies in Poland and can be used as a reference to the actual epidemiological situation prevailing in ICU units of other hospitals in Poland, Europe or other continents. The aim of the study was to determine the incidence and microbiological profile of healthcare-associated infections in an intensive care unit.

## 2. Materials and Methods

The active surveillance method was used to detect HAIs in adult patients who spent over 48 h in a 9-bed general ICU ward located in southern Poland between 2007 and 2016. The ward under study was part of a multi-profile hospital (670 hospital beds). Not all patients included in the study were examined with the application of SOFA and APACHEII scales, therefore these scales were not taken under consideration. Definitions developed by the ECDC were used to identify and qualify infections [6,7]. The data collection methodology was fully compliant with the European Centre for Disease Prevention and Control (ECDC) recommended by the Healthcare-associated Infections Surveillance Network (HAI-Net) [7]. The HAI study was divided into the main and specific types of HAIs, such as BSI, PN, UTI, and OTH (other infections). Among the OTH infections, the following were distinguished: CNS, EENT, LRI, SYS. 

Bloodstream infection was defined as a patient has at least one positive blood culture for a recognised pathogen or patient has at least one of the following signs or symptoms: fever (>38 °C), chills, or hypotension and two positive blood cultures for a common skin contaminant (from two separate blood samples, usually within 48 h). Skin contaminants = coagulase-negative staphylococci, *Micrococcus* spp., *Propionibacterium acnes*, *Bacillus* spp., *Corynebacterium* spp. [7]. 

Pneumonia (PN1–PN5) was defined as two or more serial chest X-rays or CT-scans with a suggestive image of pneumonia for patients with underlying cardiac or pulmonary disease. In patients without underlying cardiac or pulmonary disease, one definitive chest X-ray or CT-scan is sufficient. And at least one of the following: fever >38 °C with no other cause leukopenia (<4000 WBC/mm^3^) or leucocytosis (≥12,000 WBC/mm^3^) and at least one of the following (or at least two, if clinical pneumonia only = PN4 and PN5): new onset of purulent sputum, or change in character of sputum (colour, odour, quantity, consistency), cough or dyspnea or tachypnea suggestive auscultation (rales or bronchial breath sounds), rhonchi, wheezing, worsening gas exchange (e.g., O_2_ desaturation or increased oxygen requirements or increased ventilation demand) and according to the used diagnostic method [7].

Urinary tract infection (UTI) was defined as a microbiologically confirmed symptomatic urinary tract infection where the patient has at least one of the following symptoms with no other recognised cause: fever (>38 °C), urgency, frequency, dysuria, or suprapubic tenderness and patient has a positive urine culture, i.e., ≥105 microorganisms per mL of urine with no more than two species of microorganisms [7]. 

A device-associated, healthcare-associated infection is an HAI in a patient with a (relevant) device that was used within a 48-h period before the onset of infection. The term ‘deviceassociated’ is only used for pneumonia, bloodstream infections, and urinary tract infections. ‘Relevant devices’ refers to intubation, central vascular catheters, and urinary catheters. If the interval is longer than 48 h, there must be compelling evidence that the infection was associated with the device use. For catheter-associated UTI, an indwelling urinary catheter must have been in place seven days before positive laboratory results or signs and symptoms meeting the criteria for UTI were evident. Example: Pneumonia is defined as intubation-associated (IAP) if an invasive respiratory device was present (even intermittently) in the 48 h preceding the onset of infection [7]. 

Blood infections were monitored as Catheter Related Infections (CRIs) including the division into types of catheters used and microbiological diagnosis. 

The results of microbiological studies performed with clinically relevant materials, such as blood, urine, smear from wounds, faeces, broncho-aspirate, vascular lavage, vascular catheter tips and others, were interpreted. The hospital applied the principles of the European Committee on Antimicrobial Susceptibility Testing (EUCAST). Strains resistant to antibiotics were defined in the following way: Extensive Drug Resistant (XDR)—resistant to one or more antibiotics in all groups of antibiotics; multi drug resistant (MDR)—resistant to at least one antibiotic from three or more groups of antibacterial drugs active for a particular type of microbial genus or Pan Drug Resistant (PDR)—resistant to all antibiotics in all groups of antibiotics active for a particular genus. Microbial cultures were incubated on chromogenic media: Chromid ID CPS, Columbia with 5% of ram blood, Sabouraud agar, Schaedler agar, trypicase soy agar, chocolate agar, MacConkey agar, Hektoen enteric agar, sodium selenate agar. Microorganisms were identified by means of biochemical tests applied in automatic system of identification: Vitek 2 Compact (bioMérieux, Marcy-l’Étoile, France).

The following epidemiological indicators were used to investigate the epidemiological situation: cumulative incidence and incidence density. The incidence = number of HAI × 100 per number of patients. The incidence density of HAIs per 1000 days of device use. Density CLA-BSI = number of CLA-BSI × 1000 per number of central line-days. Density IAP = number of IAP × 1000 per number of intubation-days (VAP w CDC/INICC). Density CA-UTI = number of CA-UTI × 1000 per number of urinary catheter-days. Mortality (% death of HAIs = number of deaths HAI × 100 per number of HAI). Device utilization ratio (DUR) = number of an invasive device days (CVC, intubation, urinary catheter) per number of patient days. All the data that were entered into an electronic database and analyses in this study were anonymized previously.

Statistical Package for the Social Sciences (SPSS STATISTICS 24, Armonk, NY, USA) and Microsoft Excel Microsoft Office 2016 (Redmond, WA, USA) were used in the statistical analysis of the collected material. The significance level was *p* < 0.05. In addition, the pooled mean, median (Me), standard deviation (SD), 95% confidence interval for mean (95% CI), odds ratio (OR), Chi-squared (Pearson) χ^2^ for the ordinal and nominal scales and an ANOVA for the quantitative scale were calculated. The invasive procedures performed on patients were analyses as risk factors for HAI formation and calculated for Chi-squared (Pearson)’s materiality level, the quotient of chances and Pearson’s correlations.

## 3. Results

During the 2007–2016 period 1847 patients were treated in the ICU, for more than 48 h each; 710 patients (38.4%) were females, and 1137 patients (61.6%) were males. The HAI risk was statistically significantly higher for men (*p* < 0.001) than for women, and the odds of HAI (OR) were greater for men than for women and amounted to 1.55 (95% CI 1.24–1.92). The average age of patients hospitalized in the ICU was 58 (median 61 years, SD 20, and 95% CI 57–59). Patients with HAIs were younger and were, on average, 54 years old (median 57 years, SD 18.495, and 95% CI 52.8–56.1) than patients without HAIs, who were on average 59 years old (median age 62, SD 19.840, 95% CI 58.2–60.2); this difference was statistically significant (*p* < 0.001). 

In the group of 1847 patients under survey, 510 cases of HAI were diagnosed, and the HAI incidence rate was 27.6% in 100 patients admitted to the ICU. The main types of HAI detected in ICU patients included the following: PN (9.9% incidence), BSI (8.9% incidence) and UTI (2.9% incidence). The central line-associated bloodstream infection (CA-BSI) incidence density was 8.0/1000 catheter-days, the device use ratio (DUR) was 0.96, the ventilator-associated pneumonia (VAP) incidence density was 15.2/1000 ventilator-days, (DUR 0.65) and the catheter-associated urinary tract infection (CA-UTI) incidence density was 3.0/1000 catheter-days. In the material under examination, other types of HAI accounted for 22% of all infections, and the percentage of these infections was similar in all the years under analysis (Table 1).

The analysis identified risk factors, which included invasive procedures and potentially a significant factor in HAI formation. Statistically significant differences occurred between patients without and with HAIs in the following invasive procedures: mechanical ventilation, urethral catheter, administration of blood and plasma, pleural drainage, tracheostomy, percutaneous endoscopic gastrostomy (PEG), bronchoscopy, parenteral nutrition, and gastroscopy. Poor impact strength (Pearson’s correlation) was found for the administered blood, plasma, pleural drainage, PEG probes, bronchoscopy and gastroscopy, indicating that the tendency to acquire HAI is evident with the use of these devices; there are, however, deviations. Moderate dependence was detected in the case of a patient who had tracheostomy, in which case the tendency to acquire an HAI was most visible (Table 2).

A total of 93% of HAIs were caused by micro-organisms that were determined by being identified as a genus or species. A total of 7% of HAI infections were not microbiologically confirmed. BSI was dominated by Coagulase-negative staphylococci (44%), PN was dominated by non-fermenting gram-negative bacteria including *Acinetobacter baumannii* (41%), UTI was dominated by Enterobacteriaceae including *Escherichia coli* (19%), SSI was dominated by *A. baumannii* (25%), GI was dominated by *Clostridium difficile* (70%), SST was dominated by *E. coli* (20%) and *Klebsiella pneumoniae* (20%), and OTH was dominated by *K. pneumoniae* (18%) (Table 3).

The average time between admission of a patient to the ICU and emergence of HAI was 22.9 days (median 17 days) and was statistically significantly different for each type of HAI (*p* < 0.001). The average duration of an ICU patient’s stay was 28.2 days (median 17 days). Patients with HAIs stayed in the ICU statistically significantly longer than those without HAIs (*p* < 0.001) (Table 4).

The number of days that elapsed before the infection and the duration of a patient’s stay in the ICU were different for each species (or genus) of microorganism. In patients with HAIs, the mortality rate was 42.5%, and in the non-infected group, the mortality rate was 45.3%; this difference was not statistically significant (*p* = 0.296). The index calculated for each species (species) of the microorganism showed that the highest mortality rate was observed in patients with fungal infections (Table 5).

Antibiotic resistance mechanisms were identified for the most common microorganisms (Table 5). Antibiotic susceptibility analysis was performed for the three most common Gram-negative organisms. *Acinetobacter baumannii* strains were characterized by high rates of resistance to many groups of antibiotics, 87% of which were extensive-drug resistant (XDR) strains. Similarly, 25% of K. pneumoniae strains were included in XDR, and 17% were multi-drug resistant (MDR). Pseudomonas aeruginosa strains were mostly antibiotic-sensitive, with resistance affecting 30% of the strains classified as MDR strains (Table 6).

The number of HAIs caused by the *A. baumannii* strains significantly fluctuated in the years under studyexamined herein, most frequently in 2008–2009, and a rapid trend of *A. baumannii* resistance to carbapenems was observed (Table 7). 

## 4. Discussion

The results of our single-centre study are among the very few available in Poland. The study has a limited character because observational single-centre studies cannot be treated as a benchmark for all the population of Polish patients treated in ICUs. Therefore, the results of the study should be treated as an information basis on which preventive strategy of HAIs should be built accompanied by active methods of supervision aimed at improving the safety of patients treated in ICUs. It is not easy to assess the condition of a critically ill patient treated in ICU and it is not always possible because of the dynamics of pathological changes and the intensity of treatment. Thus, the next limitation for the studies is lack of proper classification of patients’ condition based on APACHEII and SOFA scale. Lack of such an evaluation in the group of 1849 patients treated in ICUs within 10 years resulted in not including the results of several hundred patients into the total analysis. That is why the study is focused mainly on evaluation of various types of HAIs and their determinants. In our ICU, the incidence of HAIs was 28%. The results obtained were similar to the results obtained in the meta-analysis performed by Allegranzi et al. [4], in which the incidence was 30%. In one of the single-centre studies conducted in Poland by Wieder-Huszla [2] in 2010, the incidence of HAIs was 24%. Additionally, Kübler et al. [3] conducted another single-centre study in Poland in 2012 and reported the incidence of HAIs as 24%. In the point prevalence survey (PPS) based on morbidity and performed in Poland in 2011–2013, morbidity in the ICU was 35%. In the literature, lower HAI incidence rates have also been reported, ranging from 9% to 16% [5,15]. In our ICU, the mortality of patients with healthcare-associated infections was 12%. The result is similar to the mortality rate reported in the 2007 ECDC report, with an average mortality rate ranging to 15% [1]. Agodi et al. [16] detected 18% mortality in the ICU examined. The average age of patients treated in the ICU was 58 years, with a predominance of men, and the ratio of men to women was 1.55. In other European countries, the average age of patients was 61, and the sex male-to-female ratio was 1.56 [1]. In Rosenthal et al. [17], the average age of patients was 60 years. This analysis indicated that the patients in the analysed ward were younger, which may imply a worse health status in the Polish population and an earlier incidence of the diseases than in the compared countries. In our study, the duration of a patient’s stay in the ICU was 19 days for patients without HAIs and as many as 55 days for patients with HAIs. In European countries, the length of ICU stays was between 8 and 13 days (the average amounting to 10 days) [1]. The extended stay of patients in the examined ward may be related to a different healthcare organization in Poland as well as to the infrastructure of the hospital wards, whose managements are afraid of accepting and continuing the treatment of a patient in a difficult state of health [18]. For patients with HAI, their longer stays may be related to the location of the infection as well as to the species of infecting micro-organism and its susceptibility to antibiotics [19,20]. 

Pneumonia was most frequently diagnosed, the incidence being 10%. In the ECDC report, which included data from 2007 [1], the average incidence was lower at 7%, but the differences between European countries were large and ranged from 3% to 36%. In a subsequent ECDC report in 2014, the average incidence was 6% [21]. In Rosnthal et al. [22] report from 50 countries the VAP incidence ranged between 0.9 and 13.1 per 1000 ventilator-days. Ventilator associated pneumonia density in the American CDC NHSN program from 2012 was on average 0.9 per 1000 ventilator-days [23]. In the present study, the IAP density was 15 in 1000 days with intubation. In European countries, in a study published in 2012, the mean density of IAP was 8 in 1000 with intubation [1]. In Rosenthal et al. [17] the IAP density in different ICUs ranged from 10 to 53 per 1000 days with a respirator. In another study of 43 countries, 17 patients were treated with a respirator [24]. In ECDC reports published in the last few years the mean device-adjusted rate was 10 IAP episodes per 1000 intubation days and varied between 3 (UK-Scotland) and 16 (Italy) in 2014; 2 (Luxembourg) and 18 (Italy) in 2015 [25,26]. In a single-centre study conducted in Poland in the ICU in 2012/2013/2014, the prevalence of a IAP was 11/9/10 for 1000 days with intubation, respectively [27]. The average time between the admission to the onset of PN in our study was 19 days (median 15 days). In an ECDC report, the mean incubation time from ICU admission to the onset of pneumonia was 16 days (median 10 days) [1]. In our study, the duration of hospitalization for PN patients was 52 days on average (median 38 days), which means a significant, almost three-fold increase in hospitalizations in comparison with that of non-infected patients. Another study conducted in Poland confirmed the long 36-day hospitalization of PN patients [28]. In the ECDC study, the duration of hospitalization of patients with PN was approximately 3.5 times higher than that of patients without PN [1]. In the further part of our own study, PN subtypes were identified (based on diagnostics), with PN4 dominating (145 cases (79%)). Similar evidence can be found in the ECDC report, in which there were approximately 50% of PN4. However, there were significant differences between the countries surveyed [1]. *A. baumannii* (41%), *P. aeruginosa* (12%), *S. aureus* and *E. coli* (9%) and *K. pneumoniae* (8%) were predominant in our study. For comparison, in the ECDC study pneumonia was most commonly caused by the following: *P. aeruginosa* (19%), *S. aureus* (16%), and *E. coli* (9%) [1]. In another ECDC study conducted in 2011–2012 for infections in European countries, PN was predominant in *Pseudomonas aeruginosa* (17%), *Staphylococcus aureus* (13%), and *Klebsiella* spp. (11%) [29]. The ECDC study found the same microorganisms in a similar proportion, the only difference being a more frequent occurrence of *Acinetobacter baumannii* in our ICU. *Acinetobacter baumannii* also predominated in PNs detected in ICU in other Polish studies [2,27]. In a study by Chmielarczyk et al. [30], *Acinetobacter baumannii* induced 79% of PN. It can therefore be concluded that similar PN aetiological factors are present in regions of Poland and other European countries. The mortality rate in our study was 45%. 

The second-most common type of HAI was bloodstream infections (BSIs), with a 9% incidence. In the 2007 ECDC report, the incidence in European countries ranged from 2% to 5% (on average 4%) [1]. In a subsequent ECDC report published in 2015, the incidence was 3% [21]. In our study, the density of catheter-associated bloodstream infections (CLA-BSI) was 8 per 1000 central-line days. As the 2012 European ECDC report reported, the density of bloodstream infections with CLA-BSI varied between 2 and 5 in 1000 central-line days in Europe (the average being 3 in 1000 CVC days) [1]. Comparing our findings with those from European programmes, it could be observed that bloodstream infections are twice as common in our study in the incidence rates and densities. In another study conducted in 55 ICUs in developing countries, the density of bloodstream infections with CLA-BSI ranged from 8 to 19 per 1000 central-line days (average of 13 per 1000 central-line days) [17]. Rosnthal et al. [22] in the report INICC 2010–2015 from 50 countries CLA-BSI ranges between 0.8 and 4.1 per 1000 central-line days. CLA-BSI density obtained in the American CDC NHSN program in 2012 was on average 1.8 per 1000 central-line days [23]. In ECDC report from 2014 the mean device-adjusted rate was: 2.4 CLA-BSI episodes per 1000 central-line days [26]. In ECDC from 2015 the mean device-adjusted rate was 3.6 CLA-BSI episodes per 1000 central-line days and varied between 1 (Luxembourg) and 8 (Slovakia) [25]. The obtained results classified the occurrence of catheter-associated bloodstream infections in Poland at the lower border of developing countries with respect to CLA-BSI prevalence. In another study of 43 countries, the density was 5 per 1000 CVC days [24]. The average time between admission to BSI was 24 days (median 16 days). In the ECDC report [1], the onset of BSIs occurred on day 20 (median 14 days). In our BSI study, the following physiological flora were dominant in BSIs: Coagulase-negative staphylococci (44%), *A. baumannii* (17%), and *S. aureus* (6%). The results obtained were comparable to those reported in the 2012 ECDC study for CLA-BSIs [1], where coagulase-negative staphylococci was present in 41%, *S. aureus* was present in 13%, and *Enterococcus* spp. was present in 12%. In the 2017 ECDC report [26], coagulase-negative staphylococci were similarly dominant. In a study by Chmielarczyk et al. [30] conducted in Poland in 2016, *Acinetobacter baumannii* was detected in 17% cases in BSI in ICU. The mortality rate for BSIs was 45%. In a study carried out in Poland by Wójkowska-Mach et al. [31], the mortality rate related to primary blood infection in ICU was higher and amounted to 19%. In the 2007 ECDC report, mortality in BSIs averaged 33% (21% to 40%) [1].

The third-most common form of hospital infection was urinary tract infections. In our study, the incidence rate was 3%, and the density of UTI with catheter-associated urinary tract infection (CA-UTI) amounted to 3 per 1000 catheter days. In a single-centre study conducted in Poland by Duszyńska et al. [32], the incidence rate was 7%. In the 2007 ECDC report, the incidence in European countries was 7%, and the density was 1 to 21 (with an average of 5 per 1000 catheter days) [1]. In Rosnthal et al. [22] INICC 2010–2015 raport from 50 countries CA-UTI ranges from 1.7 to 5.1 per 1000 catheter days. CA-UTI Density in American CDC NHSN program from 2012 was on average 1.7 per 1000 catheter days [23]. In a study conducted in 2006 in 55 intensive care units in developing countries, the incidence of CA-UTI ranged from 2 to 13 per 1000 catheter days [17]. In a study of 43 countries conducted in 2014, the density was 5 per 1000 catheter days [24]. The lower CA-UTI scores, which we obtained, may be due to the weakness of the HAI surveillance structures and little experience in this field [18]. In ECDC from 2014 and 2015 the mean device-adjusted rate was 4 CA-UTI episodes per 1000 catheter days [25,26]. In the ECDC report [1], the onset of UTI was on the twenty-first day of residence (median: 15 days). In the ICU under our examination, the onset of UTI was on average 30 days (median: 24 days), and the average hospitalization time was 62 days (median 54 days), which implies a significant increase in hospitalization for non-infected patients. In our study, UTIs was dominated by the following microbiota: *Candida albicans* (20%), *E. coli* (19%), and *P. aeruginosa* (11%). The microbiota detected in our study was similar to the one from 2007 ECDC report: *Escherichia coli* (25%), *C. albicans* (17%), and *Enterococcus* spp. (18%). The UTI mortality rate was 41%. In the European study, a large variation—between 7% and 37%—in UTI mortality rates was observed [1]. 

In the ICU in our study, other types of healthcare-associated infections were present: GI (2%), SSI (2%), SST (1%), and OTH (1%). In a multicentre prevalence-based study, healthcare-associated infections were detected in the ICU in slightly different percentages: GI (morbidity 4%), SSI (9%), SST (3%) and OTH (4%) [10].

In the present study, the prevalence’s of multidrug resistance (MDR) strains was maintained at a similar level in particular years: *Staphylococcus aureus* MRSA (21%), MRSE coagulase-negative staphylococci (36%), ESBL *Escherichia coli* (7%), *Klebsiella* spp. (23%), and *Enterococcus* spp. (31%). In the 2007 ECDC report, the findings were as follows: *Staphylococcus aureus* MRSA (35%), coagulase-negative staphylococci (83%), *E. coli* ESBL (12%), *Klebsiella* spp. (23%) [1,21], *Escherichia coli* ESBL (12%), *Klebsiella* spp. (26%), *Staphylococcus aureus* MRSA (25%), and *Enterococcus* spp. (25–50%) [21]. However, in other studies, a clear trend was observed for antimicrobial resistance, especially among Gram-negative bacilli [19,20,29]. The 2017 ECDC report [26], provided the following results: 25% *Staphylococcus aureus* MRSA, 17% ESBL *E. coli*, 44% *Klebsiella* spp., and 44% *Enterobacter* spp. 

Clinical specimens from HAI patients were isolated from multidrug resistance (MDR) strains, which implies insufficient sensitivity to at least one antibiotic from three or more antimicrobial drug classes active against the species. In our study, 30% of *Staphylococcus aureus* strains showed methicillin resistant (MRSA) strains, but no vancomycin resistant strains (VRSA) were detected. The stabilization of MRSA infections and the small percentage of Gram (+) microorganisms resistant to vancomycin-resistant bacteria has been observed in the literature [19]. A literature analysis shows that the growth in resistance to antibiotics of Gram (−), especially *K. pneumoniae* [19,20,22,26,29] is on the increase. In the present study, the percentage of *K. pneumoniae* resistant to third-generation cephalosporins ranged from 17% to 67%. In a study conducted under the PPS (ECDC) programme in Europe in 2012, *K. pneumoniae*, isolated from invasive infections, showed, on average, a 26% resistance to third-generation cephalosporins (from 2% to 79%); in Poland, *K. pneumoniae* showed a 73% resistance [29]. 

While analysing the antimicrobial resistance of antibiotics, *Acinetobacter baumannii* was found to be 87% extensive-drug resistant (XDR), which means there was insensitivity to one or more antibiotics in all but two or fewer classes of antibiotics. Based on a review of the literature and the observed trends of resistance among *Acinetobacter baumannii*, the results obtained in our own study were comparable to the results of reports from European countries, where the microorganism displayed a wide spectrum of resistance [29]. In a study by Chmielarczyk et al. [30], extensive drug resistant strains (XDR) accounted for 80.8% of the tested isolates and were detected in HAI patients in the ICU. Therefore, monitoring drug resistance of *A. baumannii* strains in ICUs is vital with respect to planning specific infection control measures to prevent the spread of MDR and XDR to other patients.

According to Różańska et al. [33], in Poland, infection control programmes are a challenge for the future, and their implementation requires increasing the awareness of both medical staff and hospital management. The presented results constitute some of the research based on the method of active, continuous surveillance of infections in the ICU in Poland. The results obtained confirm the reliability of surveillance conducted in the ICU under examination. The results can provide a basis for comparing the epidemiological situation in other ICUs, which may accordingly contribute to effective HAI prevention.

## 5. Conclusions

(1) In the ICU study, pneumonia and bloodstream infections were the most common.
(2) *Acinetobacter baumannii* strains were most commonly isolated from clinical specimens collected from HAI patients.
(3) The number of *Acinetobacter baumannii* infections did not have a tendency during the period considered.
(4) The strains of *Acinetobacter baumannii* mostly showed resistance to many antibiotic groups.
(5) There was a trend of an increasing resistance of *Acinetobacter baumannii* to carbapenems.
(6) Action is needed to limit the spread of these strains.

## Figures and Tables

**Table 1:** Number of HAIs/100 admissions (%), number of HAIs/1000 person days (*n*/1000), person days (*n*) by major and specific sites of infection in an intensive care unit in the years 2007–2016.

HAI Types	*N*	Incidence (%) per 100 Admissions to ICU	Incidence Density per 1000 Days in ICU	Number of Person Days with Invasive Device *	Incidence Density per 1000 Person Days with Device	DUR
All HAIs	510	27.6	29.0			
**Pneumonia (PN)**	**183**	**9.9**	**10.4**			
PN1: minimally contaminated LRT sample with quantitative culture	13	0.7	0.7	12,059	15.2 *	0.65
PN2: non-protected sample with quantitative culture	14	0.8	0.8			
PN3: alternative microbiological criteria	6	0.3	0.3			
PN4: sputum bacteriology or non-quantitative	145	7.9	8.2			
PN5: no microbiological documentation	5	0.3	0.3			
**Bloodstream infections (BSI)**	**159**	**8.6**	**9.0**			
BSI-C: Primary BSI catheter-related	24	1.3	1.4	17,858	8.0 **	0.96
BSI-UO: Primary BSI of unknown origin	118	6.4	6.7			
BSI-S: secondary BSI	17	0.9	1.0	n/a	n/a	n/a
**Urinary tract infection (UTI)**	**54**	**2.9**	**3.1**			
UTI-A: microbiologically confirmed symptomatic	54	2.9	3.1	18,192	3.0 ***	0.98
UTI-B: not microbiologically confirmed symptomatic	0	0,0	0.0			
**Gastrointestinal (GI)**	**38**	**2.1**	**2.2**			
GI-CDI: clostridium difficile infection	20	1.1	1.1	n/a	n/a	n/a
GI-GE: gastroenteritis	18	1.0	1.0	n/a	n/a	n/a
**Surgical site infection (SSI)**	**33**	**1.8**	**1.9**			
SSI-D: Deep incisional	18	1.0	1.0	n/a	n/a	n/a
SSI-O: Organ/space	6	0.3	0.3	n/a	n/a	n/a
SSI-S: Superficial incisional	9	0.5	0.5	n/a	n/a	n/a
**Skin and soft tissue infection (SST)**	**25**	**1.4**	**1.4**			
SST-DECU: soft tissue	17	0.9	1.0	n/a	n/a	n/a
SST-SKIN: skin infection	5	0.3	0.3	n/a	n/a	n/a
SST-ST: soft tissue	3	0.2	0.2	n/a	n/a	n/a
**Other infections (OTH)**	**18**	**1.0**	**1.0**			

Number of patients in ICU = 1847; the number of days in ICU = 17,599, HAI: hospital acquired infections; *N*: number; * per 1000 ventilator days; ** per 1000 central line days; *** per 1000 catheter days; n/a: not applicable; LRT: lower respiratory tract, device use ratio (DUR).

**Table 2:** HAI risk factors in the ICU, 2007–2016.

Risk Factor	Patients without HAI	HAI Patients	Total	OR (95% CI)	*p*	Pearson’s Correlation
	*n* (%)	*n* (%)	*n* (%)			
Central catheter	885 (72.5%)	335 (27.9%)	1220 (100%)	1.0 (0.8–1.2)	0.869	0.0
Arterial catheter	443 (71.0%)	181 (29.0%)	624 (100%)	1.1 (0.9–1.4)	0.595	0.0
Intubation	287 (86.2%)	134 (31.8%)	421 (100%)	1.3 (1.0–1.7)	0.265	0.1
Mechanical ventilation	887 (69.8%)	383 (30.2%)	1270 (100%)	1.5 (1.2–1.9)	<0.05	0.1
Urethral catheter	259 (65.9%)	134 (34.1%)	393 (100%)	1.5 (1.2–1.9)	<0.001	0.1
Stomach probe	447 (71.0%)	183 (29.0%)	630 (100%)	1.1 (0.9–1.4)	0.884	0.0
Blood	310 (53.0%)	275 (47.0%)	585 (100%)	3.9 (3.1–4.8)	<0.001	0.3
Pleural drainage	67 (46.5%)	77 (53.5%)	144 (100%)	3.4 (2.4–4.8)	<0.001	0.2
Tracheostomy	98 (27.9%)	253 (72.1%)	351 (100%)	12.4 (9.5–16.3)	<0.001	0.5
Peg	38 (30.6%)	86 (69.4%)	124 (100%)	6.9 (4.7–10.3)	<0.001	0.3
Plasma	196 (54.0%)	167 (46.0%)	363 (100%)	2.8 (2.2–3.6)	<0.001	0.2
Bronchoscopy	117 (39.1%)	182 (60.9%)	299 (100%)	5.8 (4.5–7.5)	<0.001	0.3
Parenteral nutrition	101 (57.4%)	75 (42.6%)	176 (100%)	2.1 (1.5–2.9)	<0.001	0.1
Gastroscopy	37 (26.4%)	103 (73.6%)	140 (100%)	8.9 (6.0–13.2)	<0.001	0.3

Chi-square (*p*); Odds ratio (OR); 95% confidence interval (CI); percutaneous endoscopic gastrostomy (PEG).

**Table 3:** Microorganisms isolated in HAI in the ICU, 2007–2016.

Number of HAI with Microorganisms, All	*N*	BSI	PN	UTI	SSI	GI	SST	OTH
		156	174	54	32	27	25	18
		*n* (%)	*n* (%)	*n* (%)	*n* (%)	*n* (%)	*n* (%)	*n* (%)
**Gram-positive cocci (*n*)**	**138**	**79**	**21**	**0**	**2**	**0**	**2**	**4**
Coagulase-negative staphylococci (%)	70	69 (44.2)						1 (5.6)
*Staphylococcus aureus* (%)	33	10 (6.4)	16 (9.2)		2 (6.3)		2 (8.0)	3 (16.6)
*Enterococcus* spp. (%)	32		2 (1.1)					
*Streptococcus pneumoniae* (%)	3		3 (1.7)					
**Gram-positive other (*n*)**	**19**	**0**	**0**	**0**	**0**	**19**	**0**	**0**
*Clostridium difficile* (%)	19					19 (70.4)		
**Enterobacteriaceae (*n*)**	**117**	**24**	**46**	**20**	**8**	**0**	**13**	**8**
*Escherichia coli* (%)	44	8 (5.1)	16 (9.2)	10 (18.5)	4 (12.5)		5 (20.0)	1 (5.6)
*Klebsiella pneumoniae* (%)	35	8 (5.1)	14 (8.0)	2 (3.7)	1 (3.1)		5 (20.0)	7 (17.9)
*Enterobacter* spp. (%)	18	4 (2.6)	7 (4.0)	4 (7.4)	1 (3.1)		2 (8.0)	
*Proteus* spp. (%)	11		7 (4.0)	2 (3.7)	1 (3.1)		1 (4.0)	
*Seratia* spp. (%)	5	3 (1.9)	1 (0.6)		1 (3.1)			
*Citrobacter* spp. (%)	4	1 (0.6)	1 (0.6)	2 (3.70)				
**Non-fermenting gram-negative bacteria (*n*)**	**165**	**33**	**96**	**10**	**13**	**0**	**6**	**7**
*Acinetobacter baumannii* (%)	120	27 (17.3)	72 (41.4)	4 (7.4)	8 (25.0)		4 (16.0)	5 (27.7)
*Pseudomonas aeruginosa* (%)	39	5 (3.2)	20 (11.5)	6 (11.1)	5 (15.6)		1 (4.0)	2 (11.1)
*Morganella morgannii* (%)	3	1 (0.6)	1 (0.6)				1 (4.0)	
*Stenotrophomonas maltophilia* (%)	2		2 (1.1)					
*Hemophilus* spp. (%)	1		1 (0.6)					
**Other bacteria (*n*)**	**9**	**3**	**3**	**0**	**2**	**0**	**1**	**0**
Other bacteria (%)	9	3 (1.9)	3 (1.7)		2 (6.3)		1 (4.0)	
**Fungi (*n*)**	**31**	**6**	**5**	**11**	**3**	**5**	**0**	**0**
*Candida albicans* (%)	28	6 (3.8)	5 (2.9)	11 (20.4)	1 (3.1)	5 (18.5)		
*Candida glabrata* (%)	2				2 (6.3)			
*Candida krusei* (%)	1							
**Viruses (*n*)**	**2**	**0**	**0**	**0**	**0**	**2**	**0**	**0**
Adenovirus	1					1 (3.7)		
Norovirus	1					1 (3.7)		

*N*: number; pneumonia (PN); blood stream infections (BSI); urinary tract infection (UTI); Gastrointestinal (GE); Surgical site infection (SSI); skin and soft tissue infection (SST); Other infections (OTH).

**Table 4:** Days until HAI onset, ICU length of stay, mortality related to the microorganism that gave rise to HAI in the ICU in the years 2007–2016.

Infection Types	*N*	Average Number of Days between Admission to HAI Emergence		Average Number of Days of Patient’s Stay in ICU	
		Pooled Mean (95% CI)	SD	Pooled Mean (95% CI)	SD
type HAI					
Patients without HAI	1337	n/a	n/a	19.0 (17.9–20.1)	21.1
Pneumonia	183	19.2 (16.7–21.7)	17.3	51.5 (45.8–57.2)	39.0
Blood stream infections	159	24.1 (20.2–27.9)	24.7	49.2 (42.9–55.6)	39.7
Urinary tract infection	54	30.4 (23.9–36.8)	23.6	62.3 (50.9–73.6)	41.2
Gastrointestinal	38	13.7 (11.2–16.3)	7.7	42.2 (29.8–55.0)	37.8
Surgical site infection	33	26.3 (21.4–31.2)	13.8	67.0 (50.7–83.3)	45.9
Skin and soft tissue infection	25	42.0 (29.2–54.8)	31.1	73.2 (55.7–90.6)	41.3
Other infections	18	14.3 (7.5–21.1)	13.7	34.5 (23.6–51.6)	28.2
Total	1847	22.9 (21.0024.8)	21.0	28.2 (26.8–29.7)	31.5
Materiality ANOVA (*p*)	n/a	*p* < 0.001	n/a	*p* < 0.001	n/a
selected aetiological HAI factors					
*Clostridium difficile*	19	12.4 (8.4–16.4)	8.3	35.5 (19.2–51.8)	32.7
*Staphylococcus aureus*	33	19.0 (16.1–22.0)	16.2	50.3 (33.4–80.6)	40.8
*Staphylococcus* CNS	70	20.8 (16.4–32.5)	28.5	47.5 (35.7–58.5)	39.5
*Enterobacter* spp.	18	22.7 (15.6–23.1)	15.7	58.3 (42.0–71.9)	43.3
*Acinetobacter baumannii*	120	23.6 (17.6–29.6)	19.7	40.0 (37.2–51.0)	22.7
*Escherichia coli*	44	24.3 (18.0–29.0)	15.3	53.7 (46.3–61.1)	40.6
*Klebsiella pneumoniae*	35	27.1 (13.2–37.5)	25.3	67.8 (46.4–77.8)	45.1
*Candida albicans*	28	31.5 (23.0–39.9)	26.2	57.5 (45.9–69.1)	38.3
*Pseudomonas aeruginosa*	39	35.9 (24.4–44.4)	29.7	59.7 (42.3–73.5)	42.8
*Enterococcus* spp.	32	36.2 (25.5–45.6)	30.2	66.3 (52.3–80.2)	43.1

*N*: number; Me: median; SD: standard deviation; 95% confidence interval (CI) for mean; n/a: not applicable; CNS: coagulase-negative staphylococci.

**Table 5:** Antimicrobial resistance in HAI in the ICU, 2007–2016.

Microbial Genus/Species	Number of Isolates, Total	Number of Isolates with Resistance Mechanism	Resistant Codes	
		*n* (%)		*n* (%)
Gram-positive cocci				
*Staphylococcus aureus*	33	10 (30.3)	MRSA	7 (21.1)
			MLSB	3 (9.1)
Coagulase-negative staphylococci (%)	70	30 (42.8)	MRSA	25 (35.7)
			MLSB	5 (7.1)
*Enterococcus* spp.	32	10 (31.2)	HLAR	10 (31.2)
Enterobacteriaceae				
*Escherichia coli*	44	3 (6.8)	ESBL	3 (6.8)
*Klebsiella pneumoniae*	35	10 (28.6)	MBL	2 (5.7)
			ESBL	8 (22.9)
Non-fermenting gram-negative bacteria				
*Acinetobacter baumannii.*	120	5 (4.1)	MBL	5 (4.1)
*Enterobacter* spp.	18	3 (16.6)	ESBL	3 (16.6)

MRSA: methicillin-resistant *Staphylococcus aureus*; ESBL: extended beta-lactamase produced; HLAR: high-level aminoglycoside resistance; MBL: metalo-beta lactamase.

**Table 6:** Resistance to a selected group of antibiotics and type of multi-drug resistance in the ICU in the years 2007–2016.

Antibiotic Name	*Acinetobacter baumannii* (*N* = 120)	*Pseudomonas aeruginosa* (*N* = 39)	*Klebsiella pneumoniae* (*N* = 44)
Aminoglycosides (%)			
Gentamicin	63.7	25.0	25.0
Tobramycin	81.1	10.0	33.3
Amikacin	95.6	15.0	25.0
Netilmicin	88.4	20.0	16.6
Antipseudomonal carbapenems (%)			
Imipenem	30.4	35.0	0.0
Mero penem	33.3	20.0	8.3
Extended-spectrum cephalosporins (%)			
Ceftazidime	98.1	15.0	41.6
Cefepime	94.5	25.0	16.6
Cefotaxime	99.3	75.0	58.3
Ceftriaxone	97.8	65.7	66.6
Antipseudomonal fluoroquinolones (%)			
Ciprofloxacin	97.8	10.0	41.6
Levofloxacin	95.6	10.0	8.3
Penicillins (piperacillin) (%)			
Piperacillin	0.0	20.0	0.0
Penicillins and b-lactamase inhibitors (%)			
Ticarcillin clavulanic acid	0.0	20.0	16.6
Piperacillin tazobactam	98.7	0.5	25.0
Ampicillin Sulbactam	57.8	0.0	0.0
Cephalosporins and inhibitors (%)			
Cefoperazone sulbactam	11.5	15.0	16.6
Polymyxins (%)			
Colistin	1.2	0.5	0.0
Polymyxin B	0.0	0.0	0.0
Type of resistance			
Sensitive (%)	1.4	65.0	58.3
MDR (%)	11.5	30.0	16.6
XDR (%)	86.9	0.0	25.0
PDR (%)	0.0	0.0	0.0

**Table 7:** Compilation of the number of *Acinetobacter baumannii* isolates resistant to carbapenems in the years 2007–2016.

Year	Number of *Acinetobacter baumannii* Isolates	Imipenem	Meropenem
		*n* (%)	*n* (%)
2007	11	1 (9.1)	0 (0.0)
2008	26	2 (7.7)	1 (3.8)
2009	26	3 (11.5)	2 (7.7)
2010	7	1 (14.4)	4 (57.1)
2011	3	1 (33.3)	1 (33.3)
2012	13	6 (46.2)	9 (69.2)
2013	4	1 (25.0)	3 (75.0)
2014	11	7 (63.6)	7 (63.6)
2015	13	10 (76.9)	10 (76.9)
2016	6	4 (66.7)	3 (50.0)
totality	120	36 (30.4)	40 (33.3)

*n* (%): number and percentage of strains resistant to imipenem and meropenem.

## Abbreviations

HAIs	Healthcare-Associated Infections
DA-HAI	Device-Associated Healthcare-Associated Infection
ICU	Intensive Care Unit
ECDC	European Centre for Disease Prevention and Control
PN	Pneumonia
IAP	Intubation Associated Pneumonia
VAP	Ventilator Associated Pneumonia
PN2	Non-protected sample with quantitative culture
PN3	Alternative microbiological criteria
PN4	Sputum bacteriology or non-quantitative
PN5	No microbiological documentation
BSI	Bloodstream Infection
CVC	Central Venous Catheter
CLA-BSI	Central Line-Associated Bloodstream Infection
BSI-C	Vascular Catheter-Related
CRI1-CVC	Primary BSI local Catheter Related Infection
CRI2-CVC	Primary BSI general Catheter Related Infection
CRI3-CVC	Primary BSI microbiologically confirmed Catheter Related
BSI UO	Primary BSI of Unknown Origin
BSI-S	Secondary BSI to another infection: DIG, PUL, SSI
UTI	Urinary Tract Infections
CA-UTI	Catheter-Associated UTI
UTI-A	Microbiologically confirmed symptomatic
UTI-B	Not microbiologically confirmed symptomatic urinary tract infection
GI	Gastrointestinal
GI-CDI	Clostridium difficile infection
GI-GE	Gastroenteritis
SSI	Surgical Site Infection
SSI-D	Deep incisional
SSI-O	Organ/space
SSI-S	Superficial incisional
SST	Skin and Soft Tissue Infection
SST-DECU	Decubitus ulcer
SST-SKIN	Skin infection
SST-ST	Soft tissue
CNS	Central Nervous System Infections
EENT	Eye, Ear, Nose, Throat or Mouth Infection
LRI	Lower Respiratory Tract
SYS	Systemic Infections
OTH	Other Infections
BAL	Broncheoalveolar Lavage
CFU	Colony Forming Unit
XDR	Extensive Drug Resistant
PDR	Pan Drug Resistant
MDR	Multi Drag Resistant
EUCAST	European Committee on Antimicrobial Susceptibility Testing
*p*	Significance level
Me	Median
SD	Standard Deviation
95% CI	Confidence Interval
OR	Odds ratio
DUR	Device Use Ratio
INICC	International Nosocomial Infection Control Consortium
NHSN	National Halthcare Safety Network in USA
APACHEII	Acute Physiology And Chronic Health Evaluation II score
SOFA	Sepsis-Related Organ Failure Assessment score
